# The Effects of Previous Amenorrhea on Endothelial and Vascular Function

**DOI:** 10.1155/ije/6305659

**Published:** 2025-01-27

**Authors:** Katherine T. Williford, Emma V. Frye, Rebecca M. Kappus

**Affiliations:** Department of Public Health and Exercise Science, Appalachian State University, Boone, North Carolina, USA

**Keywords:** aortic blood pressure, arterial stiffness, beta-stiffness, estrogen, flow-mediated dilation, physical activity

## Abstract

**Introduction:** Endogenous estrogen has a protective cardiovascular effect. Estrogen loss, such as during menopause and amenorrhea, results in stiffer vessels and endothelial dysfunction. It is unknown if reversing amenorrhea and regaining a regular menstrual cycle leads to the restoration of cardiovascular function.

**Methods:** Sixteen women were divided into groups: eumenorrheic (*n* = 10; 23 ± 3 years) women who had a consistent menstrual cycle and amenorrheic (*n* = 6; 22 ± 1 year) women who previously were without their menstrual cycle for at least 6 consecutive months. Endothelial function, arterial stiffness, and brachial and aortic blood pressure were assessed.

**Results:** There were no differences between groups in endothelial function or arterial stiffness measures. The previously amenorrheic group displayed lower brachial systolic and mean pressures and aortic systolic, diastolic, and mean pressures. When controlling for physical activity, only central pressure remained significantly lower in the amenorrheic group.

**Conclusions:** Previous amenorrhea in young women does not result in long-term cardiovascular consequences in arterial and endothelial function measures assuming regular menstruation resumes. Physical activity shows a blood pressure–lowering effect in the peripheral arteries, while the previously amenorrheic group demonstrated lower central pressures, independent of physical activity.

## 1. Introduction

Endogenous estrogen is considered cardioprotective due to its effect on vascular smooth muscle and endothelial regulation. Estrogen has vasodilatory actions, inhibits the migration and proliferation of smooth muscle cells, and has anti-inflammatory and vasoprotective effects [1–5]. This is reflected frequently, but not always [6, 7], in enhanced cardiovascular function in premenopausal females compared to age-matched males [8, 9] and an increased incidence of cardiovascular disease in postmenopausal females [10].

Throughout their reproductive years, women may miss a menstrual cycle unrelated to purposeful birth control methods. Secondary amenorrhea is defined as the absence of a menstrual cycle for 3 consecutive months or more following menarche. The most common types of secondary amenorrhea are pregnancy and lactation. However, secondary amenorrhea may also occur due to dysfunction with the hypothalamus. Ovarian hormones interact within the hypothalamic–pituitary–ovarian axis via gonadotropin-releasing hormone (GnRH), which regulates ovulation and reproduction [11]. The suppression of GnRH, termed functional hypothalamic amenorrhea (FHA), is characterized by a loss of menses and low estrogen [11]. Contributing factors to FHA are disordered eating, stress, low body fat, excessive exercise, or energy imbalances [11]. These factors are common among young women, especially those who are physically active, highlighting the interplay of low energy availability, amenorrhea, and osteoporosis, resulting in the genesis of the term “female athlete triad” [12]. Additionally, despite the higher levels of activity, athletes with amenorrhea do not display higher levels of lean mass when compared to athletes with eumenorrhea and inactive eumenorrheic controls but do exhibit lower bone mineral density (BMD) [13]. No matter the cause of FHA, the common pathway underlying the disruption of ovulation and resulting amenorrhea is a change in the normal pattern of secretion of GnRH [14].

Because of the known link between endogenous estrogen and cardiovascular health, it is possible that amenorrhea may be detrimental to the long-term health of the heart and blood vessels [4]. Premenopausal women who experience amenorrhea are at a higher risk of developing osteoporosis and premature cardiovascular disease [15] which highlights the need to intervene if a female experiences amenorrhea.

The normal estrogen loss that occurs with aging results in decreased nitric oxide bioavailability and subsequent endothelial dysfunction, demonstrated via a reduction in brachial flow-mediated dilation (FMD) [16]. FMD is a noninvasive assessment used to determine vasodilator function in the macrovasculature. Following an ischemic stimulus, there is a subsequent increase in blood flow and shear stress; the resulting increase in vascular diameter due to shear stress is largely indicative of nitric oxide bioavailability in the endothelium and is reflective of vascular health and future cardiovascular risk [17]. Although there are different mechanisms for the estrogen loss seen in menopause and secondary amenorrhea, women suffering from secondary amenorrhea display a similar reduction in endothelial function compared to postmenopausal women and have significantly lower brachial FMD compared to eumenorrheic women [18–21]. Further, amenorrheic women display reduced nitric oxide metabolites despite higher dietary intake [22], elevated low-density lipoprotein cholesterol, total cholesterol, and triglyceride levels [23, 24], and increased susceptibility to lipid peroxidation [25].

Research into the cardiovascular effects of amenorrhea focuses on premenopausal women who were currently amenorrheic at the time of testing. In addition, although there are many studies assessing endothelial function in amenorrheic women, there are limited data on arterial stiffness or vascular remodeling measures. It is unknown if regaining a regular menstrual cycle reverses the cardiovascular dysfunction seen in amenorrheic women and if previous amenorrhea leads to long-term cardiovascular dysfunction compared to women who never experience amenorrhea. If there are lasting detrimental effects of amenorrhea, despite resuming a regular menstrual cycle, this knowledge may result in preventative action or earlier interventions in young women.

This study sought to determine whether previously amenorrheic women demonstrate impaired cardiovascular function despite regaining a regular menstrual cycle. We hypothesize that women who have previously suffered from amenorrhea will exhibit lower endothelial function, stiffer vessels, and higher pressures compared to women without a history of amenorrhea.

## 2. Methods

### 2.1. Participants

Sixteen females between the ages of 18 and 30 years were recruited among the local university community. At the time of data collection, all participants had a regular menstrual cycle for a minimum of previous 6 months. The amenorrheic group consisted of six women who, at some time in their life, were without their menstrual cycle for at least six consecutive months, not due to factors such as birth control, medication usage, thyroid disease, pregnancy, lactation, or menopause. The eumenorrheic group consisted of 10 women who had a consistent menstrual cycle, defined as not missing a menstrual cycle for more than two consecutive months. Participants were excluded if they were pregnant or breastfeeding, smokers, or taking medication known to affect cardiovascular function, inflammation, or metabolic function in the previous 2 weeks. Participants were free of cardiovascular disease, metabolic disease, cancer, chromosomal disorders, pituitary tumors, and anomalies of the reproductive system. Prior to data collection, all participants provided written informed consent and the study was approved by the Appalachian State Institutional Review Board. All study procedures adhered to the Declaration of Helsinki.

### 2.2. Study Design

Participants reported to the lab once during the early follicular phase of their menstrual cycle. Participants reported to the lab in the morning following an overnight fast and abstained from alcohol, exercise, and caffeine at least 24 h prior to the visit. Participants filled out a health history questionnaire to determine eligibility and to assess weekly physical activity status. Maximal voluntary contraction (MVC) was measured to determine the overall muscular strength [26]. Participants performed 3 maximal handgrip contractions with a 5-min rest between each attempt, squeezing a dynamometer (TSD121C hand dynamometer, Biopac Systems, Inc., Goleta, CA). The best attempt of the three contractions was used as the MVC. Height was measured in centimeters using a stadiometer and weight was assessed using a digital scale (Health-o-meter 349KLX Medical Scale) in kilograms. Body mass index (BMI) was calculated using weight in kilograms divided by height in meters squared. Participants underwent a dual-energy X-ray absorptiometry (DEXA) scan (GE Lunar iDXA; GE Healthcare, Madison, Wisconsin, USA) for assessment of body composition, including body fat percentage (BF%) and whole-body BMD. Following these measures, participants rested supine for 10 min in a darkened, quiet, and temperature-controlled room prior to the cardiovascular measurements.

### 2.3. Peripheral (Brachial) Blood Pressure

Resting systolic and diastolic brachial blood pressure (bSBP and bDBP) were measured at the brachial artery using an automated oscillometric cuff (HEM-907 XL; Omron, Shimane, Japan). Blood pressure was taken in duplicate, and if the two values were not within 5 mmHg, additional measurements were taken until 2 values within 5 mmHg of each other were obtained. The averaged values were used for analysis. Mean arterial pressure (MAP) was calculated as bDBP + 1/3(bSBP − bDBP) and brachial pulse pressure (bPP) was calculated as bSBP − bDBP.

### 2.4. Central Pulse Wave Velocity (cfPWV)

Applanation tonometry (AtCor Medical, SphygmoCor Technology, Sydney, Australia) was used to measure carotid-femoral pulse wave velocity (cfPWV). A single high-fidelity pressure transducer was used to measure pressure waveforms at the right common carotid artery while a blood pressure cuff simultaneously measured pressure waveforms at the right femoral artery. The distance between measurement sites was measured in centimeters. The cfPWV is the difference between measurement sites and the time delay between the proximal and distal waveforms. Measurements were made in duplicate, and if not within 0.5 m/s, additional measurements were taken. The average of the two closest values was used for analysis.

### 2.5. Central (Aortic) Blood Pressure

Radial artery pressure waveforms were obtained using applanation tonometry and calibrated with the brachial blood pressure obtained previously. Using a generalized validated transfer function [17, 27], a central aortic pressure waveform is reconstructed from the radial artery pressure waveform (AtCor Medical, SphygmoCor Technology, Sydney, Australia) to obtain aortic systolic and diastolic blood pressure (aSBP and aDBP). Aortic mean arterial pressure (aMAP) and aortic pulse pressure (aPP) were determined from the integration of the reconstructed aortic pressure waveform using the SphygmoCor software. This technique has been validated for use in obtaining central pressure [27]. Carotid pressure was obtained using applanation tonometry on the carotid artery and calibrated against brachial mean and diastolic pressures. Carotid pressures were used in carotid artery stiffness assessment. Pulse wave reflection was calculated as the augmentation pressure expressed as a percentage of the aPP (augmentation index [AIx]). AIx was normalized to a heart rate of 75 bpm (AIx@75) to control for variations in heart rate.

### 2.6. Carotid Intima–Media Thickness (cIMT)

The right common carotid artery was imaged with ultrasound (Arietta 70, Aloka, Tokyo, Japan) using a 7.5-MHz linear-array probe (SSD-5500; Aloka, Tokyo, Japan) 10 to 20 mm proximal to the carotid bifurcation. The intima–media thickness of the common carotid artery, defined as the distance between the leading edge of the lumen–intima interface to the leading edge of the media–adventitia interface of the far wall of the carotid artery, was measured at the end of the diastole.

### 2.7. Carotid Artery Stiffness (β-Stiffness)

Using the same ultrasound and linear-array probe, the carotid artery was imaged again to determine the β-stiffness index, which adjusts arterial compliance for changes in distending pressure. Carotid *β*‐stiffness was calculated as a means of adjusting arterial compliance for changes in distending pressure using the following equation:

(1) $$\begin{array}{c} \text{Carotid }\beta ‐\text{stiffness}\frac{\log\;P1/P0}{\left(D1-D0/D0\right)}, \end{array}$$

where *P*1 and *P*0 are the highest (systolic) and lowest (diastolic) carotid pressures and *D*1 and *D*0 are the maximum (systolic) and minimum (diastolic) diameters.

### 2.8. Flow-Mediated Vasodilation (FMD)

FMD was assessed using ultrasonography (Arietta 70, Aloka, Tokyo, Japan). A rapid release cuff was placed below the elbow joint on the widest part of the elbow. The subject laid supine with the right arm stabilized using an immobilizer cushion. The brachial artery was captured in longitudinal image sections, 5–10 cm proximal to the placement of a blood pressure cuff, using a high-frequency linear-array probe [17]. In B-mode, split mode was used to simultaneously measure the arterial diameter and Doppler velocity. The flow signals were corrected at an insolation angle of 60 degrees. The sample volume was placed in the middle of the artery, with a large sampling area. Gated images were recorded at 5 frames per second using Vascular Tools (Medical Imaging Applications, Coralville, IA, USA). Baseline measurements of resting brachial flow velocity and diameter were assessed for 60 s, and the average flow velocity and brachial diameter were reported. Average brachial diameter (in cm) and average brachial flow velocity (in m/s) were used to calculate brachial blood flow using the equation: Flow Velocity *π*(brachial⁣diameter^2^/4) × 60. The blood pressure cuff was then inflated with an ischemic stimulus maintained for 5 min. The diameter was recorded for 3 min postdeflation and the peak diameter following cuff release was reported. A single technician collected and analyzed all FMD data. FMD% was calculated as (Peak Diameter − Baseline Diameter/Baseline Diameter) × 100.

### 2.9. Statistical Analysis

Data were analyzed using the IBM Statistical Package for Social Sciences Software for Windows, Version 29.0 (SPSS, Armonk, NY: IBM Corp). An independent *t*-test was run comparing the eumenorrheic and amenorrheic groups with descriptive variables. When significance was detected between groups, a Bonferroni post hoc analysis was performed. An ANCOVA was performed to control for variance and confounding factors. A Pearson correlation analysis was performed to determine the effects of physical activity volume on independent variables in both groups. Statistical significance was set at an alpha < 0.05. Data are presented as mean ± standard deviation.

## 3. Results

Participant characteristics are provided in Table 1. There were no differences between groups in age, height, weight, BMI, BF%, BMD, or MVC. Amenorrheic women performed more weekly physical activity, an average of 280 min/week versus the eumenorrheic group at 156 min/week. Both groups reported engaging primarily in aerobic activity, and there were no differences in muscle strength (MVC) between groups. There were no significant differences between menarche age in the eumenorrheic and amenorrheic groups. Of the 16 participants, 6 were taking birth control (2 in the eumenorrheic group and 4 in the amenorrheic group). The women who previously experienced amenorrhea had a loss of menses between 9 and 15 months (mean: 11.5 months) and regained a regular menstrual cycle between 6 and 72 months prior (mean: 33 months).

Arterial stiffness and endothelial function measures are presented in Table 2. There were no significant differences in any of these measurements between eumenorrheic and amenorrheic women.

There were significant differences between groups in bSBP, bMAP, aSBP, aDBP, and aMAP (Table 3), with the amenorrheic group displaying lower brachial and aortic pressures.

Because of the significant group differences in physical activity, a one-way ANCOVA was performed to determine the effects of physical activity on brachial and aortic blood pressures. Physical activity did not impact the significant difference between groups in aSBP (*p* = 0.03) and aMAP (*p* = 0.05) but did eliminate significance seen in bSBP, bMAP, and aDBP (adjusted means reported in Table 4).

Correlation analysis was performed between physical activity and all vascular function, arterial stiffness, and blood pressure measures in both groups. Significant correlations, found in the amenorrheic group only, are reported in Table 5. Brachial diameter, bPP, and aPP were found to be positively associated with physical activity while FMD% and HR were negatively associated with physical activity.

## 4. Discussion

This study assessed systemic vascular function (peripheral blood pressure and central blood pressure) and subclinical markers (cIMT, carotid β-stiffness, cfPWV, and FMD) for cardiovascular disease in eumenorrheic and previously amenorrheic women. There were no significant differences between groups in endothelial function or vascular stiffness measures, but amenorrheic women displayed lower bSBP, bMAP, aSBP, aDBP, and aMAP. In addition, there were no long-term effects of prior amenorrhea on BMD, as assessed via DEXA. Collectively, these findings suggest that despite a significant period of amenorrhea, females do not display permanent negative remodeling of their vasculature assuming a regular menstrual cycle has been regained. Thus, while amenorrheic and hypoestrogenic milieu is associated with endothelial dysfunction, this appears to be reversible and does not have a long-term effect on cardiovascular function.

### 4.1. Endothelial Function

Research that has established the link between estrogen and endothelial-dependent vasodilation has primarily been completed in postmenopausal women who display a consistent decline in endogenous estrogen [28]. Current research further demonstrates this link by studying amenorrheic women who exhibit low estrogen levels. Multiple studies in premenopausal women have shown that amenorrhea results in significantly lower brachial FMD and endothelial function compared to eumenorrheic women [18–22]. Thus, much of this research concerning estrogen and cardiovascular function is being performed in women who are currently experiencing low estrogen levels. It is unclear if significant estrogen loss will have a detrimental effect even despite the regaining of a menstrual cycle. Our findings demonstrated no difference in FMD in previously amenorrheic women compared to consistently eumenorrheic women.

It is worth noting the significantly higher levels of physical activity in the previously amenorrheic group (280 min/week compared to 156 min/week in the eumenorrheic group). Exercise can increase the risk of menstrual dysfunction, and a greater volume of exercise results in higher occurrences of amenorrhea and subsequent endothelial dysfunction [18, 29]. Conversely, exercise, and the associated increased blood flow and shear stress along the endothelial cells, also improves nitric oxide bioavailability [30]. Regular exercise and repeated episodes of shear stress result in improvements in endothelial function [31]. It is possible that because amenorrheic women were engaged in high levels of exercise, they were able to maintain endothelial function due to the regular bouts of shear stress. However, research has demonstrated that in postmenopausal estrogen-deficient women, endurance training results in increased FMD only in a group receiving an estradiol treatment, suggesting estrogen is essential to improvements in endothelial function, and not exercise alone [32]. This implies that rather than being able to maintain high endothelial function during their amenorrheic period, the previously amenorrheic group was able to restore endothelial function once they increased their estradiol levels and subsequently regained their menstrual cycle. This is consistent with a study that demonstrated that with proper estrogen levels and a menstrual cycle continuously maintained for 2 years, reversal of endothelial dysfunction can occur [33]. Thus, if exercise volumes are resulting in amenorrhea, the exercise itself may not be assisting with nitric oxide release. This also stresses the need to restore estradiol to appropriate levels in order to maintain endothelial health and reduce CVD risk. Therefore, endothelial dysfunction does not persist and/or can be reversible in premenopausal women who regain a menstrual cycle following amenorrhea.

### 4.2. Arterial Stiffness

Chronic endothelial dysfunction may lead to changes in vascular structure, further increasing the risk of CVD [34]. This can occur due to damage to the artery, plaque deposition, or an increase in the collagen/elastin ratio, resulting in stiffening of the arteries [35, 36]. Arterial stiffness is one of the earliest detectable signs of negative structural and functional changes in the vessel [37] and estrogen deficiency promotes adverse arterial remodeling seen via an increase in collagen synthesis and a decrease in degradation [38, 39]. Arterial stiffening is also augmented in females during menopause, signifying an increase in stiffness due to estrogen loss [40, 41]. Indeed, estrogen-deficient postmenopausal women show greater arterial stiffening compared with age-matched premenopausal women [42]. It is possible that previous amenorrhea and estrogen deficiency may have the same detrimental effects on arterial stiffness.

This study assessed several carotid and aortic stiffness measures. Carotid β-stiffness is a pressure-independent marker of carotid arterial compliance while cIMT represents the thickening of the lining of the carotid artery, which are both reflective of functional damage to the vasculature and are early markers of the atherosclerotic process [43]. Central PWV and AIx are indicative of aortic stiffness and are independent predictors of adverse cardiovascular events [44]. According to this study, there was no difference between groups in cIMT, β-stiffness, cfPWV, or AIx, highlighting that previous amenorrhea did not lead to adverse larger artery remodeling. Arterial stiffness is strongly linked with aging due to the structural and functional changes that occur over time [45], which may be a reason why arterial stiffening is regularly seen in postmenopausal women and was not observed in the younger cohort in the current study. In addition, the vascular aging process can be accelerated by disease, such as prolonged hypertension, and the females in the study exhibited normal blood pressure [46]. These findings suggest that estrogen loss independent of aging and age-related diseases may not result in large artery remodeling seen in long-term vascular aging.

### 4.3. Central and Peripheral Blood Pressures

There is a protective role of estrogen on blood pressure, resulting in a substantial increase in hypertension prevalence following menopause [47]. Peripheral, or brachial, blood pressure is a common clinical blood pressure assessment due to its ease of measurement. However, it is not indicative of aortic or central pressure, which more directly reflects the load on the heart and coronary and cerebral arteries and is more strongly linked to cardiovascular outcomes compared to brachial pressure [48, 49]. Therefore, having a clear assessment of both brachial and aortic pressure is necessary when assessing cardiovascular risk.

This study found that the previously amenorrheic group displayed lower central and peripheral blood pressures when compared to the eumenorrheic group, specifically bSBP, bMAP, aSBP, aDBP, and aMAP. Prior studies have shown conflicting results regarding blood pressure and estrogen. Some studies have found no differences in central and peripheral blood pressures between amenorrheic and eumenorrheic women [50, 51] while others have shown lower pressures in amenorrheic and estrogen-deficient women [52, 53].

In the current study, the lower pressures in the amenorrheic group may be attributed to their higher physical activity levels, as there is a blood pressure–lowering effect of exercise [54]. The exercise-induced vasodilation that occurs due to shear stress persists into the postexercise period, resulting in postexercise hypotension and an overall reduction in the blood pressure set point [55]. Females also have greater parasympathetic modulation following exercise compared to males [56], which results in lower heart rates and blood pressures. Thus, a one-way ANCOVA was employed to determine if these group differences in blood pressure were attributed to the differing physical activity levels. Physical activity in minutes per week did not impact the significant difference between groups in aSBP and aMAP but did eliminate significance seen in aDBP, bSBP, and bMAP, although bSBP was trending (*p* = 0.055) (adjusted means reported in Table 4). Therefore, although physical activity did play a role in the lower brachial pressures, the amenorrheic group nevertheless exhibited lower central pressures.

A Pearson correlation analysis was also performed to determine the relationship between physical activity and all variables. Only the amenorrhea group displayed significant correlations (shown in Table 5). Interestingly, the negative correlation between physical activity and FMD% suggests that higher levels of physical activity volume, in minutes per week, are correlated with reduced endothelial function. This was an unexpected finding, as increased physical activity tends to result in higher FMD% [57]. However, the reduced FMD% is likely attributed in part to the higher resting brachial diameter in the amenorrhea group [58], which is expected arterial remodeling due to exercise training [59]. In addition, endothelial function responses to exercise vary based on training type and intensity [60], which was not controlled for in this study and required further evaluation in future studies.

The negative correlation in resting heart rate, well established in regularly exercising individuals [61], is an unsurprising finding in the group with more reported physical activity. Additionally, the higher pulse pressure, reflective of an increased stroke volume in young, healthy individuals, further reflects the known effects of regular physical activity [62]. These results emphasize the role of physical activity in optimizing cardiovascular health via reductions in blood pressure and resting heart rate.

Women with exercise-associated amenorrhea display lower resting heart rate and systolic blood pressure compared with their similarly trained eumenorrheic exercising counterparts [52, 63]. This suggests that factors other than exercise could contribute to the lower values, although controlling for exercise in this study did eliminate the group differences in brachial blood pressure. However, the previously amenorrheic group displayed significantly lower central pressures independent of physical activity. The mechanism behind this is unclear, but because central blood pressure has shown to be more affected by sex, race, and factors such as pharmacological interventions [64–66], central pressure may have been more sensitive to the previous amenorrhea. Another theory is that the higher physical activity levels may have contributed to a negative energy balance, and thus, amenorrhea. Negative energy balance plays a role in reducing vagal tone in animals, resulting in lower heart rate and blood pressure [67, 68].

### 4.4. Limitations

Because of the discrepancies between groups in physical activity, it would have been ideal to match physical activity levels between groups. This would help to elucidate if consistent eumenorrhea in the presence of high physical activity volume results in better cardiovascular function than athletic women who were previously amenorrheic.

We also did not directly measure estrogen or confirm ovulation status; therefore, it is plausible that some participants may have experienced early ovulatory bleeding or breakthrough that can sometimes occur, impacting vascular measures and estradiol concentrations [18]. In addition, women taking oral contraceptives were not excluded from this study. However, all participants were tested during their early follicular stage of their menstrual cycle, or during the placebo pill phase, in order to control for sex hormone levels during testing.

Another limitation to consider is that the vascular health of the previously amenorrheic women was unknown during their amenorrheic phase. While unlikely, it is possible there were no vascular alterations, causing no significant difference to be found during the time of the current testing.

## 5. Conclusion

In the current study, previously amenorrheic women demonstrated no differences in arterial stiffness or endothelial function compared to a eumenorrheic group who had maintained a consistent menstrual cycle. The lower brachial blood pressure in the amenorrheic group was attributed to their higher physical activity levels, whereas the lower central pressure in the amenorrheic group was found to be independent of physical activity. This research highlights that previous amenorrhea experienced early in life may not have long-term negative cardiovascular repercussions as long as regular menstruation resumes.

## Figures and Tables

**Table 1:** Descriptive characteristics.

	Eumenorrheic	Amenorrheic	*p* value	*F* value
Age (yrs)	23 ± 3	22 ± 1	0.78	0.08
Height (cm)	166.5 ± 5.5	166.2 ± 5.6	0.92	0.1
Weight (kg)	61.9 ± 9.0	62.8 ± 6.2	0.83	0.5
BMI (kg/m^2^)	22.3 ± 2.6	22.8 ± 2.4	0.70	0.16
BF (%)	28.7 ± 4.3	27.8 ± 2.8	0.65	0.21
BMD (g/cm^2^)	1.16 ± 0.08	1.17 ± 0.08	0.87	0.03
MVC (kg)	14.8 ± 1.6	14.5 ± 3.0	0.84	0.4
PA (min/wk)⁣^∗^	156 ± 71	280 ± 30	**0.001**	16.07
Age of menarche (yrs)	12 ± 1	13 ± 1	0.44	0.64

*Note:* Data are presented as mean ± standard deviation. Bold values indicate significant *p* values.

Abbreviations: BF%, body fat percentage; BMD, bone mineral density; BMI, body mass index; MVC, maximum voluntary contraction; PA, self-reported physical activity.

⁣^∗^*p* < 0.05.

**Table 2:** Arterial stiffness and endothelial function measures.

	Eumenorrheic	Amenorrheic	*p* value	*F* value
cIMT (mm)	0.49 ± 0.05	0.49 ± 0.04	0.86	0.03
β-Stiffness (AU)	4.19 ± 0.97	4.48 ± 0.68	0.53	0.41
cfPWV (m/s)	5.1 ± 0.5	4.6 ± 0.7	0.18	1.9
AIx@75 (%)	−1.20 ± 9.03	−6.17 ± 11.64	0.35	0.92
Brachial diameter (mm)	3.17 ± 0.27	3.27 ± 0.24	0.50	0.6
Avg. flow velocity (m/s)	17.6 ± 4.3	13.5 ± 2.6	0.06	4.2
FBF (mL/min)	84.3 ± 27.8	69.87 ± 21.2	0.30	1.18
MaxBD (mm)	3.52 ± 0.4	3.77 ± 0.2	0.20	2.1
FMD (%)	10.9 ± 4.4	15.3 ± 5.9	0.10	2.9
HR (bpm)	60 ± 6	52 ± 9	0.07	3.8

*Note:* Data are presented as mean ± standard deviation. β-stiffness; carotid beta stiffness, AIx@75; augmentation index at a heart rate of 75 bpm.

Abbreviations: cfPWV, carotid-femoral pulse wave velocity; cIMT, carotid intima media thickness; FBF, forearm blood flow; FMD%, flow-mediated dilation percentage; HR, heart rate; MaxBD, maximum brachial diameter.

**Table 3:** Brachial and aortic blood pressures.

	Eumenorrheic	Amenorrheic	*p* value	*F* value
bSBP⁣^∗^ (mmHg)	116 ± 7	106 ± 10	**0.03**	5.49
bDPB (mmHg)	66 ± 6	60 ± 7	0.09	3.32
bMAP⁣^∗^ (mmHg)	82 ± 5	75 ± 6	**0.02**	6.57
bPP (mmHg)	50 ± 8	46 ± 11	0.47	0.54
aSBP⁣^∗^ (mmHg)	100 ± 7	89 ± 6	**0.01**	10.9
aDBP⁣^∗^ (mmHg)	67 ± 7	59 ± 6	**0.03**	6.10
aMAP⁣^∗^ (mmHg)	82 ± 6	73 ± 4	**0.01**	13.7
aPP (mmHg)	33 ± 7	30 ± 6	0.40	0.70

*Note:* Data are presented as mean ± standard deviation. Bold values indicate significance (significant *p* values).

Abbreviations: aDBP, aortic diastolic blood pressure; aMAP, aortic mean arterial pressure; aPP, aortic pulse pressure; aSBP, aortic systolic blood pressure; bDBP, brachial diastolic blood pressure; bMAP, brachial mean arterial pressure; bPP, brachial pulse pressure; bSBP, brachial systolic blood pressure.

⁣^∗^*p* < 0.05.

**Table 4:** Adjusted means after controlling for physical activity.

	Eumenorrheic	Amenorrheic	*p* value
bSBP (mmHg)	117 ± 3	104 ± 5	0.055
bMAP (mmHg)	82 ± 2	76 ± 3	0.18
aSBP⁣^∗^ (mmHg)	100 ± 3	88 ± 4	**0.03**
aDBP (mmHg)	66 ± 2	62 ± 3	0.34
aMAP⁣^∗^ (mmHg)	82 ± 2	73 ± 3	**0.05**

*Note:* Data are presented as mean ± standard deviation. Bold values indicate significance (significant *p* values).

Abbreviations: aDBP, aortic diastolic pressure; aMAP, aortic mean arterial pressure; aSBP, aortic systolic blood pressure; bMAP, brachial mean arterial pressure; bSBP, brachial systolic blood pressure.

⁣^∗^*p* < 0.05.

**Table 5:** Significant correlations with physical activity (amenorrhea group).

	Pearson correlation (*r*)	Significance (*p*) two-tailed
Brachial diameter (mm)	0.831	**0.041**
FMD (%)	−0.813	**0.049**
HR (bpm)	−0.891	**0.017**
bPP (mmHg)	0.824	**0.044**
aPP (mmHg)	0.853	**0.031**

*Note:* Bold values indicate significance (significant *p* values).

Abbreviations: aPP, aortic pulse pressure; bPP, brachial pulse pressure; FMD, flow-mediated dilation; HR, heart rate.

## Data Availability

The data that support the findings of this study are available on reasonable request from the corresponding author. The data are not publicly available due to privacy or ethical restrictions.

## References

[1] Bakir S., Mori T., Durand J., Chen Y. F., Thompson J. A., Oparil S. (2000). Estrogen-induced Vasoprotection Is Estrogen Receptor Dependent-Evidence from the Balloon-Injured Rat Carotid Artery Model. *Circulation*.

[2] Bhalla R. C., Toth K. F., Bhatty R. A., Thompson L. P., Sharma R. V. (1997). Estrogen Reduces Proliferation and Agonist-Induced Calcium Increase in Coronary Artery Smooth Muscle Cells. *American Journal of Physiology-Heart and Circulatory Physiology*.

[3] Li G. H., Chen Y. F., Greene G. L., Oparil S., Thompson J. A. (1999). Estrogen Inhibits Vascular Smooth Muscle Cell-dependent Adventitial Fibroblast Migration In Vitro. *Circulation*.

[4] Mendelsohn M. E., Karas R. H. (1999). The Protective Effects of Estrogen on the Cardiovascular System. *New England Journal of Medicine*.

[5] Miller A. P., Feng W. G., Xing D. Q. (2004). Estrogen Modulates Inflammatory Mediator Expression and Neutrophil Chemotaxis in Injured Arteries. *Circulation*.

[6] Shenouda N., Priest S. E., Rizzuto V. I., MacDonald M. J. (2018). Brachial Artery Endothelial Function Is Stable across a Menstrual and Oral Contraceptive Pill Cycle but Lower in Premenopausal Women Than in Age-Matched Men. *American Journal of Physiology-Heart and Circulatory Physiology*.

[7] Shenouda N., Skelly L. E., Gibala M. J., MacDonald M. J. (2018). Brachial Artery Endothelial Function Is Unchanged after Acute Sprint Interval Exercise in Sedentary Men and Women. *Experimental Physiology*.

[8] Hopkins N. D., Dengel D. R., Stratton G. (2015). Age and Sex Relationship with Flow-Mediated Dilation in Healthy Children and Adolescents. *Journal of Applied Physiology*.

[9] Jensen-Urstad K., Johansson J. (2001). Gender Difference in Age-Related Changes in Vascular Function. *Journal of Internal Medicine*.

[10] Sullivan J. M., Fowlkes L. P. (1996). The Clinical Aspects of Estrogen and the Cardiovascular System. *Obstetrics & Gynecology*.

[11] Gordon C. M. (2010). Functional Hypothalamic Amenorrhea. *New England Journal of Medicine*.

[12] Nattiv A., Loucks A. B., Manore M. M., Sanborn C. F., Sundgot-Borgen J., Warren M. P. (2007). American College of Sports Medicine Position Stand. The Female Athlete Triad. *Medicine & Science in Sports & Exercise*.

[13] Christo K., Prabhakaran R., Lamparello B. (2008). Bone Metabolism in Adolescent Athletes with Amenorrhea, Athletes with Eumenorrhea, and Control Subjects. *Pediatrics*.

[14] Liu J. H., Patel B., Collins G., Feingold K. R., Anawalt B., Blackman M. R. (2000). *Central Causes of Amenorrhea*.

[15] Rothman M. S., Wierman M. E. (2013). Amenorrhea. *Endocrine Secrets*.

[16] Moreau K. L., Hildreth K. L., Klawitter J., Blatchford P., Kohrt W. M. (2020). Decline in Endothelial Function across the Menopause Transition in Healthy Women Is Related to Decreased Estradiol and Increased Oxidative Stress. *Geroscience*.

[17] Thijssen D. H., Black M. A., Pyke K. E. (2011). Assessment of Flow-Mediated Dilation in Humans: a Methodological and Physiological Guideline. *American Journal of Physiology-Heart and Circulatory Physiology*.

[18] Augustine J. A., Lefferts W. K., Dowthwaite J. N., Brann L. S., Brutsaert T. D., Heffernan K. S. (2016). Subclinical Atherosclerotic Risk in Endurance-Trained Premenopausal Amenorrheic Women. *Atherosclerosis*.

[19] Hoch A. Z., Papanek P., Szabo A., Widlansky M. E., Schimke J. E., Gutterman D. D. (2011). Association between the Female Athlete Triad and Endothelial Dysfunction in Dancers. *Clinical Journal of Sport Medicine*.

[20] O'Donnell E., Goodman J. M., Mak S., Harvey P. J. (2014). Impaired Vascular Function in Physically Active Premenopausal Women with Functional Hypothalamic Amenorrhea Is Associated with Low Shear Stress and Increased Vascular Tone. *The Journal of Cinical Endocrinology and Metabolism*.

[21] Rickenlund A., Eriksson M. J., Schenck-Gustafsson K., Hirschberg A. L. (2005). Amenorrhea in Female Athletes Is Associated with Endothelial Dysfunction and Unfavorable Lipid Profile. *Journal of Clinical Endocrinology and Metabolism*.

[22] Stacey E., Korkia P., Hukkanen M. V., Polak J. M., Rutherford O. M. (1998). Decreased Nitric Oxide Levels and Bone Turnover in Amenorrheic Athletes with Spinal Osteopenia. *Journal of Clinical Endocrinology and Metabolism*.

[23] Friday K. E., Drinkwater B. L., Bruemmer B., Chesnut C., Chait A. (1993). Elevated Plasma Low-Density Lipoprotein and High-Density Lipoprotein Cholesterol Levels in Amenorrheic Athletes: Effects of Endogenous Hormone Status and Nutrient Intake. *Journal of Clinical Endocrinology and Metabolism*.

[24] Tegg N. L., Myburgh C., O'Donnell E., Kennedy M., Norris C. M. (2024). Impact of Secondary Amenorrhea on Cardiovascular Disease Risk in Physically Active Women: A Systematic Review and Meta-Analysis. *Journal of the American Heart Association*.

[25] Ayres S., Baer J., Subbiah M. T. (1998). Exercised-induced Increase in Lipid Peroxidation Parameters in Amenorrheic Female Athletes. *Fertility and Sterility*.

[26] Lee S. Y. (2021). Handgrip Strength: An Irreplaceable Indicator of Muscle Function. *Ann Rehabil Med*.

[27] Holland D. J., Sacre J. W., McFarlane S. J., Coombes J. S., Sharman J. E. (2008). Pulse Wave Analysis Is a Reproducible Technique for Measuring Central Blood Pressure during Hemodynamic Perturbations Induced by Exercise. *American Journal of Hypertension*.

[28] Taddei S., Virdis A., Ghiadoni L. (1996). Menopause Is Associated with Endothelial Dysfunction in Women. *Hypertension*.

[29] Baranauskas M. N., Freemas J. A., Carter S. J., Blodgett J. M., Pedlar C. R., Bruinvels G. (2023). Amenorrhea and Oligomenorrhea Risk Related to Exercise Training Volume and Intensity: Findings from 3705 Participants Recruited via the STRAVA Exercise Application. *Journal of Science and Medicine in Sport*.

[30] Padilla J., Simmons G. H., Bender S. B., Arce-Esquivel A. A., Whyte J. J., Laughlin M. H. (2011). Vascular Effects of Exercise: Endothelial Adaptations beyond Active Muscle Beds. *Physiology*.

[31] Hambrecht R., Adams V., Erbs S. (2003). Regular Physical Activity Improves Endothelial Function in Patients with Coronary Artery Disease by Increasing Phosphorylation of Endothelial Nitric Oxide Synthase. *Circulation*.

[32] Moreau K. L., Stauffer B. L., Kohrt W. M., Seals D. R. (2013). Essential Role of Estrogen for Improvements in Vascular Endothelial Function with Endurance Exercise in Postmenopausal Women. *Journal of Clinical Endocrinology and Metabolism*.

[33] Hoch A. Z., Jurva J. W., Staton M. A. (2007). Athletic Amenorrhea and Endothelial Dysfunction. *Wisconsin Medical Journal*.

[34] Mitchell G. F., Vita J. A., Larson M. G. (2005). Cross-sectional Relations of Peripheral Microvascular Function, Cardiovascular Disease Risk Factors, and Aortic Stiffness: the Framingham Heart Study. *Circulation*.

[35] Ben-Shlomo Y., Spears M., Boustred C. (2014). Aortic Pulse Wave Velocity Improves Cardiovascular Event Prediction: an Individual Participant Meta-Analysis of Prospective Observational Data from 17,635 Subjects. *Journal of the American College of Cardiology*.

[36] van Sloten T. T., Schram M. T., van den Hurk K. (2014). Local Stiffness of the Carotid and Femoral Artery Is Associated with Incident Cardiovascular Events and All-Cause Mortality: the Hoorn Study. *Journal of the American College of Cardiology*.

[37] Cavalcante J. L., Lima J. A., Redheuil A., Al-Mallah M. H. (2011). Aortic Stiffness: Current Understanding and Future Directions. *Journal of the American College of Cardiology*.

[38] Sokolis D. P., Dimitriou C. A., Lelovas P., Kostomitsopoulos N. G., Dontas I. A. (2017). Effect of Ovariectomy and Sideritis Euboea Extract Administration on Large Artery Mechanics, Morphology, and Structure in Middle-Aged Rats. *Biorheology*.

[39] Zhang Y., Stewart K. G., Davidge S. T. (2000). Estrogen Replacement Reduces Age-Associated Remodeling in Rat Mesenteric Arteries. *Hypertension*.

[40] Nagai Y., Earley C. J., Kemper M. K., Bacal C. S., Metter E. J. (1999). Influence of Age and Postmenopausal Estrogen Replacement Therapy on Carotid Arterial Stiffness in Women. *Cardiovascular Research*.

[41] Smulyan H., Asmar R. G., Rudnicki A., London G. M., Safar M. E. (2001). Comparative Effects of Aging in Men and Women on the Properties of the Arterial Tree. *Journal of the American College of Cardiology*.

[42] Westendorp I. C., Bots M. L., Grobbee D. E. (1999). Menopausal Status and Distensibility of the Common Carotid Artery. *Arteriosclerosis, Thrombosis, and Vascular Biology*.

[43] Kappus R. M., Fahs C. A., Smith D. (2014). Obesity and Overweight Associated with Increased Carotid Diameter and Decreased Arterial Function in Young Otherwise Healthy Men. *American Journal of Hypertension*.

[44] Nichols W. W. (2005). Clinical Measurement of Arterial Stiffness Obtained from Noninvasive Pressure Waveforms. *American Journal of Hypertension*.

[45] Lee H. Y., Oh B. H. (2010). Aging and Arterial Stiffness. *Circulation Journal*.

[46] Renna N. F., de Las Heras N., Miatello R. M. (2013). Pathophysiology of Vascular Remodeling in Hypertension. *International Journal of Hypertension*.

[47] Ashraf M. S., Vongpatanasin W. (2006). Estrogen and Hypertension. *Current Hypertension Reports*.

[48] Roman M. J., Devereux R. B., Kizer J. R. (2007). Central Pressure More Strongly Relates to Vascular Disease and Outcome Than Does Brachial Pressure: the Strong Heart Study. *Hypertension*.

[49] McEniery C. M., Cockcroft J. R., Roman M. J., Franklin S. S., Wilkinson I. B. (2014). Central Blood Pressure: Current Evidence and Clinical Importance. *European Heart Journal*.

[50] Zeni Hoch A., Dempsey R. L., Carrera G. F. (2003). Is There an Association between Athletic Amenorrhea and Endothelial Cell Dysfunction?. *Medicine & Science in Sports & Exercise*.

[51] De Souza M. J., Williams N. I. (2004). Physiological Aspects and Clinical Sequelae of Energy Deficiency and Hypoestrogenism in Exercising Women. *Human Reproduction Update*.

[52] O'Donnell E., Harvey P. J., Goodman J. M., De Souza M. J. (2007). Long-term Estrogen Deficiency Lowers Regional Blood Flow, Resting Systolic Blood Pressure, and Heart Rate in Exercising Premenopausal Women. *American Journal of Physiology. Endocrinology and Metabolism*.

[53] Warren M. P. (1999). Health Issues for Women Athletes: Exercise-Induced Amenorrhea. *Journal of Clinical Endocrinology and Metabolism*.

[54] Shephard R. J., Balady G. J. (1999). Exercise as Cardiovascular Therapy. *Circulation*.

[55] Swain R. A., Harris A. B., Wiener E. C. (2003). Prolonged Exercise Induces Angiogenesis and Increases Cerebral Blood Volume in Primary Motor Cortex of the Rat. *Neuroscience*.

[56] Kappus R. M., Ranadive S. M., Yan H. (2015). Sex Differences in Autonomic Function Following Maximal Exercise. *Biology of Sex Differences*.

[57] Ashor A. W., Lara J., Siervo M. (2015). Exercise Modalities and Endothelial Function: a Systematic Review and Dose-Response Meta-Analysis of Randomized Controlled Trials. *Sports Medicine*.

[58] Maruhashi T., Soga J., Fujimura N. (2013). Relationship between Flow-Mediated Vasodilation and Cardiovascular Risk Factors in a Large Community-Based Study. *Heart*.

[59] Dinenno F. A., Tanaka H., Monahan K. D. (2001). Regular Endurance Exercise Induces Expansive Arterial Remodelling in the Trained Limbs of Healthy Men. *The Journal of Physiology*.

[60] Shivgulam M. E., Liu H., Schwartz B. D. (2023). Impact of Exercise Training Interventions on Flow-Mediated Dilation in Adults: An Umbrella Review. *Sports Medicine*.

[61] Reimers A. K., Knapp G., Reimers C. D. (2018). Effects of Exercise on the Resting Heart Rate: A Systematic Review and Meta-Analysis of Interventional Studies. *Journal of Clinical Medicine*.

[62] McDonnell B. J., Maki-Petaja K. M., Munnery M. (2013). Habitual Exercise and Blood Pressure: Age Dependency and Underlying Mechanisms. *American Journal of Hypertension*.

[63] O'Donnell E., Harvey P. J., De Souza M. J. (2009). Relationships between Vascular Resistance and Energy Deficiency, Nutritional Status and Oxidative Stress in Oestrogen Deficient Physically Active Women. *Clinical Endocrinology*.

[64] Heffernan K. S., Jae S. Y., Wilund K. R., Woods J. A., Fernhall B. (2008). Racial Differences in Central Blood Pressure and Vascular Function in Young Men. *American Journal of Physiology-Heart and Circulatory Physiology*.

[65] Sharman J. E., Laurent S. (2013). Central Blood Pressure in the Management of Hypertension: Soon Reaching the Goal?. *Journal of Human Hypertension*.

[66] Picone D. S., Stoneman E., Cremer A. (2023). Sex Differences in Blood Pressure and Potential Implications for Cardiovascular Risk Management. *Hypertension*.

[67] Herlihy J. T., Stacy C., Bertrand H. A. (1992). Long-term Calorie Restriction Enhances Baroreflex Responsiveness in Fischer 344 Rats. *American Journal of Physiology-Heart and Circulatory Physiology*.

[68] Williams T. D., Chambers J. B., Henderson R. P., Rashotte M. E., Overton J. M. (2002). Cardiovascular Responses to Caloric Restriction and Thermoneutrality in C57BL/6J Mice. *American Journal of Physiology-Regulatory, Integrative and Comparative Physiology*.

[69] Williford K. M. (2020). *The Effects of Previous Amenorrhea on Vascular Function*.

